# Synthesis, Characterisation and Antifungal Activities of Some New Copper(II) Complexes of Isomeric 3,5,7,7,10,12,14,14-Octamethyl-1,4,8,11-Tetraazacyclotetradecanes

**DOI:** 10.1155/MBD.1997.255

**Published:** 1997

**Authors:** Saroj K. S. Hazari, Tapashi G. Roy, Benu K. Dey, Suvash C. Das, Edward R. T. Tiekink

**Affiliations:** 1 Department of Chemistry University of Chittagong Chittagong 4331 Bangladesh; 2 Department of Chemistry The University of Adelaide 5005 Australia

## Abstract

Three isomeric Me_8_[14]anes, L_A_, L_B_ and L_C_, undergo complexation with copper(II) salts to form a series of [CuLX_n_(H_2_O)_x_]X_y_.(H_2_O)_z_ complexes where L = L_A_, L_B_ and L_C_; X = Cl, Br, NO_3_; n, x, y and z may have values of 0, 1 or 2. The complexes have been characterised on the basis of analytical, spectroscopic, magnetic and conductance data. Further, the X-ray crystal structure of one complex, [CuL_B_(OH_2_)_2_](NO_3_)_2_, has been determined. The antifungal activity of all three isomeric ligands and their complexes has been investigated against a range of phytopathogenic fungi.


### SYNTHESIS, CHARACTERISATION AND ANTIFUNGAL ACTIVITIES OF SOME NEW COPPER(II) COMPLEXES OF ISOMERIC 3,5,7,7,10,12,14,14-OCTAMETHYL-1,4,8,11-TETRAAZACYCLOTETRADECANES

Saroj K.S. Hazari[1], Tapashi G. Roy*[1], Benu K. Dey[1], Suvash C. Das[1] and Edward R.T. Tiekink*[2]

[1] Department of Chemistry, University of Chittagong, Chittagong-4331, Bangladesh
[2] Department of Chemistry, The University of Adelaide, Australia 5005

**Abstract**
Three isomeric $Me_8$[14]anes, $L_A$, $L_B$ and $L_C$, undergo complexation with copper(II) salts to form a series of $[CuLX_n(H_2O)_x]X_y.(H_2O)_z$ complexes where L = $L_A$, $L_B$ and $L_C$; X = Cl, Br, $NO_3$; n, x, y and z may have values of 0, 1 or 2. The complexes have been characterised on the basis of analytical, spectroscopic, magnetic and conductance data. Further, the X-ray crystal structure of one complex, $[CuL_B(OH_2)_2](NO_3)_2$, has been determined. The antifungal activity of all three isomeric ligands and their complexes has been investigated against a range of phytopathogenic fungi.

### 1. Introduction

The importance of synthetic macrocycle complexes is well recognised and hardly needs elaboration. This contribution focuses on the synthesis and characterisation of a series of copper(II) complexes of isomeric octamethyl tetraazatetradecanes. It has been shown that 1,2-propanediamine condenses with acetone stereospecifically to yield only the 3,10-C-meso isomer of the macrocycle 3,5,7,7,10,12,14,14-octamethyl-1,4,8,11-tetraazacyclotetradeca-4,11-diene, $Me_8$[14]diene, $L_1$; this assignment is based on $^1H$ NMR [1, 2] and has been confirmed by X-ray crystallography [3].

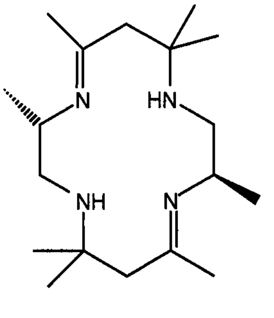

$L_1$

The reduction of $L_1$ with $NaBH_4$ yields three isomeric $Me_8$[14]anes, i.e. $L_A$, $L_B$ and $L_C$, as revealed by a $^1H$ NMR study and, in the case of $L_B$, by an X-ray crystallographic study [4]. The interactions of these ligands with certain metal centers have been investigated previously.

In one study [5], a number of square planar copper(II) complexes were prepared by the reaction, in methanolic solution, of copper perchlorate with $L_A$, $L_B$ and $L_C$; in each case two diastereoisomers were isolated. Owing to the steric hindrance of the eight methyl groups in these macrocycles and the non-coordinating tendency of perchlorate, it was expected that the preparation of five- or six-coordinate complexes may be difficult [5]. Subsequently, in another study, Bembi and co-workers [6] reported the preparation of a series of six coordinate cobalt(III) complexes with these isomeric ligands, i.e. $[CoLCl_2](ClO_4)$; N-chiral isomers have been separated. Hence, it seemed likely that higher coordination number copper(II) salts could also be prepared. In this context, a number of new four- and six-coordinate copper(II) complexes have been isolated and their antifungal activities, as well as those of the ligands, investigated.

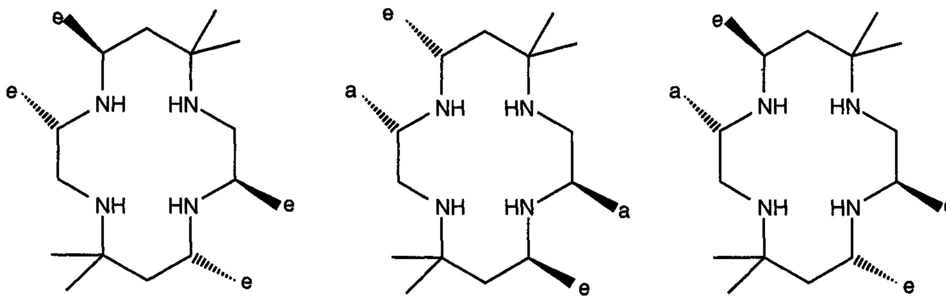

$L_A$ $L_B$ $L_C$

### 2. Experimental

### *2.1 Synthesis*

The parent ligand, 3,10-C-meso-$Me_8$[14]diene.$2HClO_4$, was synthesised according to the literature method [1] and reduction of this ligand with $NaBH_4$ was carried out in a 1:1 water-methanol mixture. The isomers, $L_A$, $L_B$ and $L_C$ were isolated by fractional crystallisation from xylene [4].

#### *2.1.1 Copper(II) nitrato complexes*

$[CuL_A(NO_3)_2].H_2O$ — $L_A$ (0.312 g, 1.0 mmol) and $Cu(NO_3)_2.3H_2O$ (0.241 g, 1.0 mmol) were dissolved separately in hot MeOH (20 ml) and EtOH (20 ml), respectively and mixed while hot. The solution was heated on a steam bath and after *ca* 30 min a brown precipitate formed. The solution was cooled and the brown product, $[CuL_A(NO_3)_2].H_2O$, was filtered off, washed with absolute EtOH and then $Et_2O$, dec. pt 256 - 258 °C. Found C, 41.76; H, 8.14; N, 16.25 %. $C_{18}H_{42}CuN_6O_7$ requires C, 41.74; H, 8.12; N, 16.24 %.

$[CuL_B(H_2O)_2](NO_3)_2$ — $L_B$ (0.312 g, 1.0 mmol) and $Cu(NO_3)_2.3H_2O$ (0.241 g, 1.0 mmol) were dissolved separately in hot dry EtOH (30 ml) and mixed slowly while hot. The solution was heated on a steam bath and after *ca* 30 min the solution was filtered. The purple filtrate was concentrated on a steam bath for a further 25 min until the volume was reduced to *ca* 15 ml. On cooling, the purple product, $[CuL_B(H_2O)_2](NO_3)_2$, crystallised. This was filtered off, washed with dry EtOH followed by $Et_2O$, and finally dried *in vacuo*. M. pt > 360 °C. Found C, 40.34; H, 8.22; N, 15.71 %. $C_{18}H_{44}CuN_6O_8$ requires C, 40.33; H, 8.22; N, 15.69 %.

$[CuL_C(NO_3)(H_2O)]NO_3$ — $L_C$ (0.312 g, 1.0 mmol) and $Cu(NO_3)_2.3H_2O$ (0.241 g, 1.0 mmol) were dissolved in dry EtOH (40 ml). The resulting blue mixture was heated on a steam bath for *ca* 30 min and then filtered. The purple filtrate was concentrated on a steam bath for a further 25 min until the volume was reduced to 10 ml. After cooling to room temperature, the blue product, $[CuL_C(NO_3)(H_2O)]NO_3$, was filtered off, washed with $^i$PrOH and $Et_2O$. The product was recrystallised from an acetonitrile solution of the complex; m. pt > 280 °C. Found C, 41.73; H, 8.13; N, 16.25 %. $C_{18}H_{42}CuN_6O_7$ requires C, 41.74; H, 8.12; N, 16.24 %.

#### *2.1.2 Copper(II) chloro complexes*

$[CuL_ACl_2].H_2O$ — $L_A$ (0.312 g, 1.0 mmol) and $CuCl_2.2H_2O$ (0.171 g, 1.0 mmol) were dissolved separately in hot dry MeOH (20 ml) and EtOH (20 ml), respectively and mixed while hot. The reaction mixture was heated on a water bath for 30 min during which time the solution changed colour from green to blue-violet. On heating for a further 30 min, the volume was reduced to *ca* 8 ml and a brick-red precipitate formed. The product, $[CuL_ACl_2].H_2O$, was filtered off, washed with absolute EtOH followed by dry $Et_2O$ and dried *in vacuo*. Further concentration of the filtrate gave a second crop which was identical with the first, m. pt 270 - 273 °C (dec.). Found C, 46.56; H, 9.02; N, 12.08 %. $C_{18}H_{42}CuCl_2N_4O$ requires C, 46.54; H, 9.04; N, 12.06 %.

$[CuL_B]Cl_2.2H_2O$ and $[CuL_BCl_2].2H_2O$ — $L_B$ (0.312 g, 1.0 mmol) and $CuCl_2.2H_2O$ (0.171 g, 1.0 mmol) were dissolved separately in hot absolute EtOH (20 ml) and mixed. The resulting violet solution was heated on a steam bath for *ca* 50 min. when a violet product began to form at high temperature. Excess $Et_2O$ was added to precipitate out the violet product, $[CuL_BCl_2].2H_2O$; m. pt > 280 °C (dec.). Found C, 44.71; H, 9.14; N, 11.62 %. $C_{18}H_{44}CuCl_2N_4O_2$ requires C, 44.72; H, 9.12; N, 11.61 %.

After separating the violet product, the violet mother liquor was concentrated to *ca* 5 ml. On cooling, a brown product precipitated. After 45 min, the product, $[CuL_B]Cl_2.2H_2O$, was filtered off and washed with dry EtOH, followed by $Et_2O$ and dried *in vacuo*; m. pt > 280 °C. Found C, 44.73; H, 9.11; N, 11.63 %. $C_{18}H_{44}CuCl_2N_4O_2$ requires C, 44.72; H, 9.12; N, 11.61 %.

The brown product turns violet when heated in an oven at 70°C for *ca* 5 min. On exposure to air the violet product reverts back to brown.

$[CuL_C]Cl_2$ and $[CuL_CCl_2].2H_2O$ — $L_C$ (0.312 g, 1.0 mmol) and $CuCl_2.2H_2O$ (0.171 g, 1.0 mmol) were dissolved separately in hot absolute EtOH (15 ml) and mixed. The resulting deep blue solution was heated on a steam bath for *ca* 45 min to reduce the volume to *ca* 20 ml. After cooling, a brown product $[CuL_C]Cl_2$ was filtered, washed with $(CH_3)_2CHOH$ and then $Et_2O$; m. pt 272 °C. Found C, 48.39; H, 8.96; N, 12.52 %. $C_{18}H_{40}CuCl_2N_4$ requires C, 48.38; H, 8.96; N, 12.55 %.

The deep blue filtrate was concentrated to *ca* 5 ml. $Et_2O$ was added in excess to precipitate the blue product, $[CuL_CCl_2].2H_2O$, which was filtered off; m. pt 272 °C. Found C, 44.75; H, 9.11; N, 11.59 %. $C_{18}H_{44}CuCl_2N_4O_2$ requires C, 44.72; H, 9.12; N, 11.61 %.

*2.1.3 Copper(II) bromo complexes*

$[CuL_ABr_2].H_2O$ — $L_A$ (0.312 g, 1.0 mmol) and $CuBr_2$ (0.223 g, 1.0 mmol) were dissolved separately in hot MeOH (30 ml) and absolute EtOH (20 ml), respectively and mixed while hot. The resulting blue solution was heated on a water bath for *ca* 65 min until the volume was reduced to *ca* 10 ml. After cooling to room temperature, dark violet crystals of $[CuL_ABr_2].H_2O$, were filtered off, washed with dry EtOH followed by $Et_2O$ and dried *in vacuo*. M. pt > 280 °C. Found C, 39.04; H, 7.60; N, 10.10 %. $C_{18}H_{42}Br_2CuN_4O$ requires C, 39.03; H, 7.59; N, 10.12 %.

$[CuL_B]Br_2.2H_2O$ and $[CuL_BBr_2].H_2O$ — A hot ethanolic solution of $L_B$ (0.312 g, 1.0 mmol, 25 ml) was added to a hot ethanolic solution of $CuBr_2$ (0.223 g, 1.0 mmol, 25 ml). The resulting purple solution was concentrated to *ca* 8 ml by heating on a steam bath for 1 h. On cooling to room temperature, a brown product, $[CuL_B]Br_2.2H_2O$, was filtered off, washed with EtOH and then dried *in vacuo*; m. pt > 280 °C. Found C, 37.81; H, 7.70; N, 9.78 %. $C_{18}H_{44}Br_2CuN_4O_2$ requires C, 37.80; H, 7.70; N, 9.81 %.

A portion of the brown product obtained above was converted to a violet product, $[CuL_BBr_2].H_2O$, by heating it in an oven at 70°C for 5 min; m. pt > 280 °C. Found C, 39.02; H, 7.58; N, 10.11 %. $C_{18}H_{42}Br_2CuN_4O$ requires C, 39.03; H, 7.59; N, 10.12 %. On exposure to air, the violet complex reverts back to the brown species.

$[CuL_CBr_2].2H_2O$ — $L_C$ (0.312 g, 1.0 mmol) and $CuBr_2$ (0.223 g, 1.0 mmol) were dissolved separately in hot absolute EtOH (15 ml) and mixed. A blue-violet color appeared immediately. The mixture was evaporated to dryness on a steam bath The crude, dried product was extracted with chloroform. Some red product remained undissolved but there was insufficient for characterisation. The chloroform extract was taken to dryness on a steam bath to yield a blue product, $[CuL_CBr_2].2H_2O$; m. pt > 280 °C. Found C, 37.82; H, 7.71; N, 9.79 %. $C_{18}H_{44}Br_2CuN_4O_2$ requires C, 37.80; H, 7.70; N, 9.81 %.

*2.2 Structure determination of $[CuL_B(H_2O)_2](NO_3)_2$*

Crystals of $[CuL_B(H_2O)_2](NO_3)_2$ were grown by the slow evaporation of an acetonitrile solution of the complex. Intensity data for a red crystal (0.40 x 0.44 x 0.44 mm) were measured at 200 K on a Rigaku AFC6R diffractometer fitted with MoKα radiation (graphite monochromator, $\lambda$ = 0.71073 Å) using the $\omega$:2$\theta$ scan technique so that $\theta_{max}$ was 27.5°. No decomposition of the crystal occurred during the data collection and the data set was corrected for Lorentz and polarization effects [7], and for absorption employing an empirical procedure (range of transmission factors: 0.882 - 1) [8]. A total of 3183 data (2988 unique) were collected and of these, 2219 that satisfied the $I \geq 3.0\sigma(I)$ criterion were used in the subsequent analysis.

*Crystal data:* $C_{18}H_{44}CuN_6O_8$, $M$ = 536.1, monoclinic, space group $P2_1/c$, $a$ = 8.100(3) Å, $b$ = 15.331(2) Å, $c$ = 10.427(3) Å, $\beta$ = 106.97(2)°, $V$ = 1238.5(6) Å$^3$, $Z$ = 2, $D_{expt}$ = 1.438 g cm$^{-3}$, $F$(000) = 574, $\mu$ = 19.35 cm$^{-1}$.

The structure was solved by placing copper at a centre of inversion and refined by a full-matrix least-squares procedure based on *F* [7]. The non-hydrogen atoms were refined with anisotropic displacement parameters and hydrogen atoms were included in the model in their calculated positions (C-H 0.97 Å); the O-H atoms were located from a difference map and included in the model. The refinement was continued until convergence with sigma weights when $R = 0.039$ and $R_w = 0.046$. The maximum residual in the final difference map was 0.44 e $Å^{-3}$. The numbering scheme employed is shown in Fig. 1 which was drawn with ORTEP [9] at 50 % probability ellipsoids. Data manipulation were performed with the teXsan program [7] installed on an Iris Indigo work station. Other crystallographic details, comprising fractional atomic coordinates for all atoms, thermal parameters, all bond distances and angles (in CIF format), and tables of observed and calculated structure factors are available on request (ERTT).

### *2.3 Physical measurements*

Visible spectra were recorded on a Shimadzu UV-visible spectrophotometer. Conductance measurements were carried out on a conductivity bridge model PW 9501 with a Phillips PW 9515/10 conductivity cell at 25 ± 0.1 °C. Magnetic measurements were made on a Sherwood Scientific magnetic susceptibility balance which was calibrated using $[HgCo(SCN)_4]$. IR spectra were recorded on a Perkin-Elmer model-883 infrared spectrophotometer as KBr disks. C, H, N analysis were carried out at the Chemistry Department, University of Stirling, Stirling, U.K.

### *2.4 Antifungal activities*

The antifungal activity of the isomeric ligands and their copper complexes (*in vitro*) against some selected phytopathogenic fungi was assessed by the poisoned food technique. Potato Dextrose Agar (PDA) was used as a growth medium. DMF was used as solvent, initially to prepare solutions of the compounds. The solutions were then mixed with the sterilised PDA to maintain the concentration of the compounds at 0.01 %; 20 ml of these were each poured into a petri dish. After the medium had solidified, a 5 mm myceial disc for each fungus was placed in the centre of each assay plate against the control. Linear growth of the fungus was measured in mm after five days of incubation at 25 ± 2°C.

### 3. Results and discussion

On the basis of their $^1H$ and $^{13}C$ NMR spectra [4], $L_A$, $L_B$ and $L_C$ have been assigned structures as shown in the *Introduction*; and the structure of $L_B$ confirmed by X-ray crystallography [4]. On reaction with copper(II) salts, the isomeric ligands yield both four- and six-coordinate complexes of the general formula: $[CuLX_n(H_2O)_x]X_y.(H_2O)_z$, where L = $L_A$, $L_B$ and $L_C$; X = Cl, Br, $NO_3$; n, x, y and z may have values of 0, 1 or 2. As $^1H$ NMR could not be measured for these paramagnetic salts, exact stereochemistries could not be determined except for that of $[CuL_B(H_2O)_2](NO_3)_2$ for which a single crystal structure analysis could be undertaken. Characterisation of the complexes could be achieved using IR and UV/vis spectroscopies as well as by magnetochemical and conductance measurements. Physical and spectroscopic data are collected in Tables 1 and 2.

In principle [6, 10], owing to the presence of four chiral N-centers in $L_A$, $L_B$ and $L_C$, each isomer $Me_8$[14]ane can yield 16 diastereoisomeric complexes of the same geometry. Out of these possibilities, only a few are stable and sufficiently abundant to permit their isolation in the solid state. In this study, only one diastereoisomer of each complex was isolated.

### *3.1 Copper(II) nitrate complexes*

Reaction of an ethanolic solution of copper(II) nitrate with $L_A$, $L_B$ and $L_C$ produce brown $[CuL_A(NO_3)_2].H_2O$, purple $[CuL_B(H_2O)_2](NO_3)_2$, and blue $[CuL_C(NO_3)(H_2O)]NO_3$, respectively.

The IR spectrum of $[CuL_A(NO_3)_2].H_2O$ exhibits a band at 1380 $cm^{-1}$, similar to that found in the spectrum of the free ligand [11], which can be assigned to absorptions due to $CH_3$ groups. Two bands, at 1435 $cm^{-1}$ and 1325 $cm^{-1}$, are attributed to coordinated $NO_3$ groups. The separation of 110 $cm^{-1}$ between the two bands indicates a unidentate mode of coordination. Moreover, a band at 250 $cm^{-1}$ can be assigned to M-O stretching of a unidentate $NO_3$ group [12]. The presence of lattice water is indicated by the presence of bands at 3440 $cm^{-1}$ and 1626 $cm^{-1}$. Selected IR bands for all complexes are collected in Table 1. The conductance value at 9 $ohm^{-1}$ $cm^2$ $mol^{-1}$ (see Table

**TABLE 1.** Selected IR bands (KBr optics, $cm^{-1}$)

| Complex | $\nu_{NH}$ | $\nu_{CH}$ | $\nu_{CH_3}$ | $\nu_{CC}$ | $\nu_{MN}$ | $\nu_{OH}$ | $\nu_{HOH}$ | other bands |
|---|---|---|---|---|---|---|---|---|
| $[CuL_A(NO_3)_2].H_2O$ | 3150 w | 2975 m | 1380 vs | 1180 s | 530 w | 3440 br | 1625 vs | 1440 ms, 1325 m, $\nu(NO_3)$ 265 s, $\nu_{MO}$ |
| $[CuL_B(H_2O)_2](NO_3)_2$ | 3195 br | 2970 m | 1380 vs | 1185 w | 540 br | 3430 br | | 1380 vs, $\nu(NO_3)$, 445 m, $\nu_{MO}$ |
| $[CuL_C(NO_3)(H_2O)]NO_3$ | 3200 br | 2965 m | 1380 vs | 1180 m | 520 sh | 3400 br | | 1440 sh, 1320 m, $\nu(NO_3)$ 245 s, 440 w, $\nu_{MO}$ |
| $[CuL_ACl_2].H_2O$ | 3135 m | 2970 s | 1360 s | 1160 s | 556 w | 3330 m | 1650 v | 275 m, $\nu_{MCl}$ |
| $[CuL_B]Cl_2.2H_2O$ | 3165 br | 2980 s | 1380 s | 1180 br | 570 sh | 3440 br | 1620 s | |
| $[CuL_BCl_2].2H_2O$ | 3160 br | 2975 s | 1370 m | 1180 w | 540 w | 3460 br | 1620 s | 240 s, $\nu_{MCl}$ |
| $[CuL_C]Cl_2$ | 3125 s | 2970 s | 1380 s | 1180 s | 530 w | | | |
| $[CuL_CCl_2].2H_2O$ | 3180 s | 2965 s | 1380 s | 1180 s | 540 sh | 3450 br | 1625 s | 250 s, $\nu_{MCl}$ |
| $[CuL_ABr_2].H_2O$ | 3160 w | 2965 m | 1380 m | 1180 s | 535 w | 3375 br | 1620 br | 245 s, $\nu_{MBr}$ |
| $[CuL_B]Br_2.2H_2O$ | 3163 s | 2970 s | 1380 s | 1183 s | 530 sh | 3460 br | 1615 s | |
| $[CuL_BBr_2].H_2O$ | 3120 s | 2960 s | 1375 s | 1180 s | 530 w | 3460 br | 1620 w | 260 s, $\nu_{MBr}$ |
| $[CuL_CBr_2].2H_2O$ | 3185 m | 2965 s | 1380 s | 1180 s | 530 sh | 3360 br | 1625 br | 530 sh, $\nu_{MBr}$ |

**TABLE 2.** Physical appearance, electronic spectral, magnetic and conductivity data

| Complex | Color in solid state | *d*-*d* bands: solvent | *d*-*d* bands: $\lambda_{max}$ (nm) (log $\varepsilon_{max}$)$^a$ | *d*-*d* bands: color in DMF | molar conductivity: ohm$^{-1}$ cm$^2$ mol$^{-2}$ | molar conductivity: color in water | molar conductivity: ohm$^{-1}$ cm$^2$ mol$^{-2}$ | magnetic moment $\mu_{eff}$ in BM |
|---|---|---|---|---|---|---|---|---|
| $[CuL_A(NO_3)_2].H_2O$ | brown | DMF<br>water | 558 (1.86)<br>516 (2.07) | brown-<br>yellow | 9 | pink | 180 | 1.80 |
| $[CuL_B(H_2O)_2](NO_3)_2$ | purple | Nujol<br>DMF<br>water | 536<br>518 (2.10)<br>511 (2.04) | pink-<br>violet | 80 | pink | 154 | 1.76 |
| $[CuL_C(NO_3)(H_2O)]NO_3$ | blue | Nujol<br>DMF<br>water | 536<br>531 (2.28)<br>523 (2.22) | blue | 83 | violet | 175 | 1.84 |
| $[CuL_ACl_2].H_2O$ | brick-red | DMF<br>water | 546 (2.08)<br>515 (1.98) | brick-red | 6 | pink | 168 | 1.97 |
| $[CuL_B]Cl_2.2H_2O$ | brown | DMF<br>water | 561 (2.08)<br>509 (2.07) | brown-<br>violet | 14 | pink | 178 | 1.64 |
| $[CuL_BCl_2].2H_2O$ | violet | Nujol<br>DMF<br>water | 536<br>551 (2.08)<br>509 (2.07) | brown-<br>violet | 9 | pink | 169 | 1.86 |
| $[CuL_C]Cl_2$ | brown | DMF<br>water | 580 (2.18)<br>519 (1.98) | brown-<br>violet | 32 | violet | 163 | 1.72 |
| $[CuL_CCl_2].2H_2O$ | blue | DMF<br>water | 581 (2.30)<br>628 (2.20) | blue | 17 | violet | 176 | 1.91 |
| $[CuL_ABr_2].H_2O$ | dark-<br>violet | DMF<br>water | 537 (1.94)<br>520 (1.08) | voliet | 23 | pink | 144 | 1.88 |
| $[CuL_B]Br_2.2H_2O$ | brown | DMF<br>water | 527 (2.09)<br>500 (2.09) | brown-<br>violet | 36 | pink | 163 | 1.67 |
| $[CuL_BBr_2].H_2O$ | violet | Nujol<br>DMF<br>water | 536<br>529 (2.14)<br>498 (1.98) | violet | 23 | pink | 155 | 2.04 |
| $[CuL_CBr_2].2H_2O$ | violet | Nujol<br>DMF<br>water | 536<br>570 (2.24)<br>529 (2.24) | brown-<br>violet | 31 | violet | 176 | 2.06 |

*a* $\varepsilon_{max}$ = maximum molar extinction coefficient in $dm^3$ $mol^{-1}$ $cm^{-1}$

2) in DMF solution shows that the complex is essentially a non-electrolyte, however, in water a 1:2 electrolyte is indicated as $H_2O$ replaces $NO_3^-$ in the coordination sphere.

It has been shown that copper(II) centres in macrocycles generally have square planar or tetragonally distorted octahedral geometries and that these give rise to broad bands in the visible region due to overlap of $A_{1g} \rightarrow B_{1g}$, $B_{2g} \rightarrow B_{1g}$ and $E_g \rightarrow B_{1g}$ transitions [13]. The $[CuL_A(NO_3)_2].H_2O$ complex shows a broad *d-d* band at 558 nm in DMF and 516 nm in water (Table 2) consistent with the above. The magnetic moment is 1.80 BM (Table 2), i.e. consistent with the copper(II) complex having one unpaired electron.

The IR spectrum of $[CuL_B(H_2O)_2](NO_3)_2$ exhibits an intense, sharp band at 1380 $cm^{-1}$ which is attributed to ionic, non-coordinating $NO_3$ and methyl groups. A sharp $\nu_{OH}$ band at 3430 $cm^{-1}$ is due to coordinated water and further evidence for this assignment is found in a band at 445 $cm^{-1}$ which is attributed to M-O stretching. The conductance in water shows a 1:2 electrolyte, however, in DMF, where the colour changes to pink-violet, the conductance value is indicative of an 1:1 electrolyte. This result is accounted for by $NO_3$ coordinating the copper center in DMF solution as has been seen in similar systems [14]. The magnetic moment and electronic data are consistent with an octahedral structure. Unambiguous structure determination has been afforded by a crystallographic analysis.

The molecular structure of the cation in $[CuL_B(H_2O)_2](NO_3)_2$ is shown in Fig. 1 and selected geometric parameters are listed in Table 3.

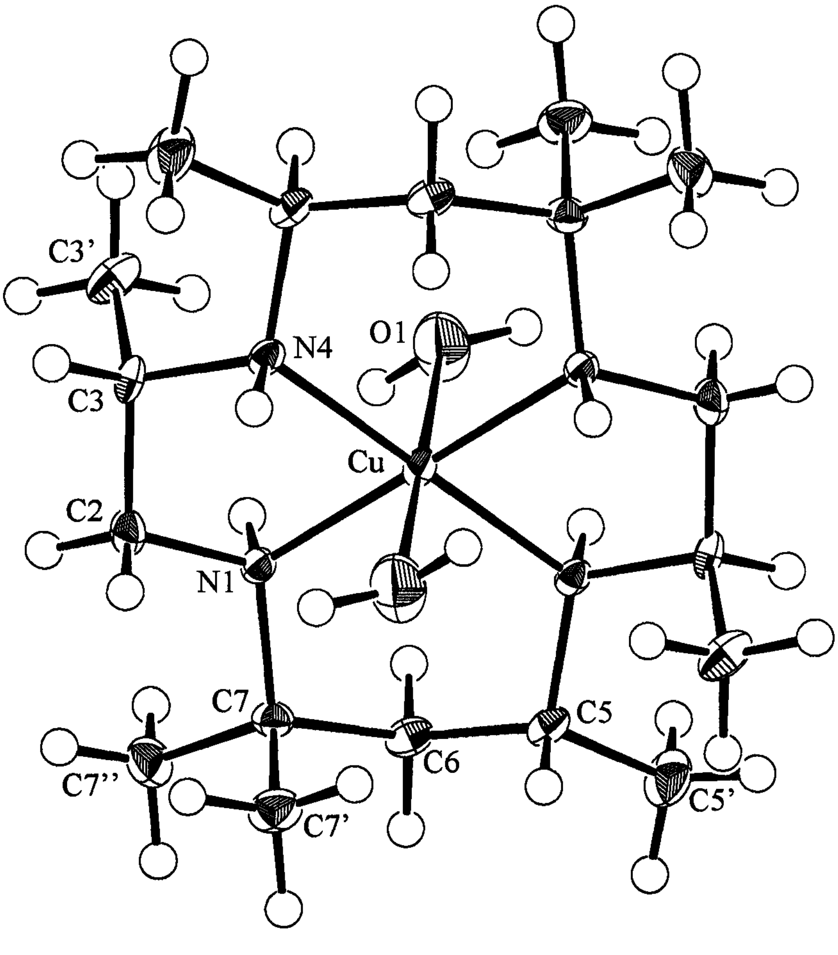

**Figure 1.** The molecular structure of the cation in $[CuL_B(H_2O)_2](NO_3)_2$ showing the numbering scheme employed.

The copper(II) cation is located on a crystallographic center of inversion and exists in a tetragonally distorted octahedral geometry defined by a $N_4O_2$ donor set. The Cu-N(1) and Cu-N(4) separations of 2.039(2) Å and 2.029(2) Å, respectively are equal to each other and the independent Cu-O(1) separation is 2.853(2) Å. The four N-chiral centers of 14-membered ring are in the 1*RS*, 4*RS*, 8*SR* 11*SR* configuration with two N<u>H</u> groups above the $N_4$ equatorial plane and the other two below. The methyl groups of the five-membered rings occupy axial positions and those in the six-membered rings, i.e. bound to C(5), occupy equatorial positions. The geometry reported here resembles closely that found in $[CuL_B(H_2O)_2](ClO_4)_2$ where the Cu-N distances were 2.035(3) Å and 2.031(4) Å, and Cu-O is 2.815(5) Å; the configuration of the four N-chiral centers was 1*SR*, 4*RS*, 8*SR*, 11*SR* with a similar disposition of the N<u>H</u> groups [15]. Trans configurations as shown in Fig. 1 have been shown to be the more stable in related systems [6, 16, 17].

**TABLE 3.** Selected interatomic parameters (Å, deg.) for $[CuL_B(H_2O)_2](NO_3)_2$. Primed atoms are related by a crystallographic center of inversion

| | | | |
|---|---|---|---|
| Cu-O(1) | 2.853(2) | Cu-N(1) | 2.039(2) |
| Cu-N(4) | 2.029(2) | N(1)-C(2) | 1.482(3) |
| N(1)-C(7) | 1.508(3) | N(4)-C(3) | 1.488(3) |
| N(4)-C(5) | 1.496(3) | C(2)-C(3) | 1.512(4) |
| C(3)-C(3') | 1.520(4) | C(5)-C(5') | 1.526(4) |
| C(5)-C(6) | 1.520(4) | C(6)-C(7) | 1.531(4) |
| C(7)-C(7') | 1.525(4) | C(7)-C(7") | 1.534(4) |
| N(5)-O(2) | 1.232(3) | N(5)-O(3) | 1.257(3) |
| N(5)-O(4) | 1.213(3) | | |
| O(1)-Cu-N(1) | 78.90(7) | O(1)-Cu-N(1)' | 101.10(7) |
| O(1)-Cu-N(4) | 106.21(7) | O(1)-Cu-N(4)' | 73.79(7) |
| N(1)-Cu-N(4) | 85.42(8) | N(1)-Cu-N(4)' | 94.58(8) |
| Cu-N(1)-C(2) | 105.9(2) | Cu-N(1)-C(7) | 119.4(1) |
| C(2)-N(1)-C(7) | 114.2(2) | Cu-N(4)-C(3) | 108.2(2) |
| Cu-N(4)-C(5) | 120.8(2) | C(3)-N(4)-C(5) | 114.6(2) |
| O(2)-N(5)-O(3) | 120.0(3) | O(2)-N(5)-O(4) | 119.5(3) |
| O(3)-N(5)-O(4) | 120.5(3) | | |

As expected there are significant hydrogen bonding interactions in the lattice of $[CuL_B(H_2O)_2](NO_3)_2$. The primary contacts involve the coordinated water molecules and the nitrate anions; weaker interactions involving the NH groups are also evident, however, such contacts are restricted owing to steric crowding. The O-<u>H</u>(1o) atom forms a contact to $O(2)^i$ such that $H(1o)...O(2)^i$ is 1.89 Å, $O(1)...O(2)^i$ is 2.853(4) Å and $O(1)-H(1o)...O(2)^i$ is 170° (symmetry operation *i*: - x, 0.5 + y, 0.5 - z) and a weaker contact to $O(4)^i$, i.e. $H(1o)...O(4)^i$ is 2.37 Å, $O(1)...O(4)^i$ is 3.109(4) Å and $O(1)-H(1o)...O(4)^i$ is 132°. The O-<u>H</u>(2o) atom is separated by 1.95 Å from $O(3)^{ii}$ with $O(1)...O(3)^{ii}$ 2.811(3) Å and $O(1)-H(2o)...O(3)^{ii}$ 152 ° (symmetry operation *ii*: - x, - y, 1 - z). The O(4) atom of the nitrate that only forms a relatively weak interaction with the water molecule forms an additional contact to the N(4)-<u>H</u>(4) atom such that $O(4)...H(4)^{iii}$ is 2.40 Å, $O(4)...N(4)^{iii}$ is 3.203(3) Å and $O(4)...H(4)^{iii}-N(4)^{iii}$ is 142° (symmetry operation *iii*: x, 0.5 - y, 0.5 + z).

In the IR spectrum of the blue $[CuL_C(NO_3)(H_2O)](NO_3)$ complex, a distinct peak at 1380 $cm^{-1}$ is found which has been assigned to overlapping ionic nitrate and methyl absorptions. Further, a medium band at 1325 $cm^{-1}$ and a shoulder at 1440 $cm^{-1}$ are indicative of coordinated $NO_3$. The separation of these bands by 115 $cm^{-1}$ and the appearance of a single sharp M-O band at 250 $cm^{-1}$ is indicative of unidentate $NO_3^-$. A band at 3400 $cm^{-1}$ shows the presence of coordinated water. The conductance at 83 $ohm^{-1}$ $cm^2$ $mol^{-1}$ in DMF solution fully supports the above assignment. By contrast, in water, where the colour changed to violet, the conductance (175 $ohm^{-1}$ $cm^2$ $mol^{-1}$) clearly suggests that $NO_3^-$ has been forced out of the coordination sphere by a $H_2O$ water. The electronic spectral and magnetic data are in good agreement with the tetragonally distorted octahedral structure, Table 2.

*3.2 Copper(II) chloro complexes*
The interaction of copper(II) chloride with $L_A$ in ethanolic solution yielded brick red $[CuL_ACl_2].H_2O$ during the course of the synthesis. A blue solution yielded this product and this suggested that a different diastereoisomer or geometric isomer was abundant in solution but only the brick-red product was stable in the solid state.

The IR spectrum of $[CuL_ACl_2].H_2O$ displays νOH and δOH bands corresponding to lattice water. A band at 275 $cm^{-1}$ is assigned to Cu-Cl stretching. The molar conductivity in DMF (6 $ohm^{-1}$ $cm^2$ $mol^{-1}$) shows that the complex is a non-electrolyte. Based on the above evidence, a distorted octahedral geometry is proposed for $[CuL_ACl_2].H_2O$.

The interaction of $L_B$ with copper(II) chloride gives brown $[CuL_B]Cl_2.2H_2O$ and violet $[CuL_BCl_2].2H_2O$. The brown product is obtained at room temperature and the violet product can be isolated in the absence of water or by heating the brown product to 70 - 80°C. Moreover, the violet complex reverts to the brown one on exposure to moisture.

The IR spectrum of brown $[CuL_B]Cl_2.2H_2O$ reveals a sharp νOH and δOH bands indicating lattice water; the absence of any M-O band around 450 $cm^{-1}$ confirms that water is not coordinated in this complex. Further, no bands are seen around 250 $cm^{-1}$ indicating that the chloride is not coordinating. The electronic spectrum was not well resolved.

The IR spectrum of violet $[CuL_BCl_2].2H_2O$ shows a similar pattern to that found for $[CuL_B]Cl_2.2H_2O$ except for the appearance of an additional band at 240 $cm^{-1}$ which is assigned to a M-Cl stretching frequency. The non-electrolytic nature of this complex in DMF solution strongly supports a tetragonally distorted octahedral complex.

An almost violet colour is observed when the brown complex is dissolved in DMF solution and the conductance (14 $ohm^{-1}$ $cm^2$ $mol^{-1}$) corresponds to a non-electrolyte. This observation suggests that in DMF solution, the non-coordinating chloride ions of $[CuL_B]Cl_2.2H_2O$ are forced into the coordination sphere. No suitable solvent was found in which the brown product remained unchanged.

Similar behaviour to that described above for $L_B$ was found for reactions involving $L_C$ where brown $[CuL_C]Cl_2$ and blue $[CuL_CCl_2].2H_2O$ were characterised.

*3.3 Copper(II) bromo complexes*
The ligand $L_A$ reacts with copper(II) bromide to yield the dark violet complex, $[CuL_ABr_2].H_2O$. With the same salt, $L_B$ produces brown $[CuL_B]Br_2.2H_2O$ which, on heating at 70°C, is converted to violet $[CuL_BBr_2].H_2O$. The two products were found to be interconvertible as found for the related chloro complexes. Blue $[CuL_CBr_2].2H_2O$ was isolated from the reaction of $CuBr_2$ with $L_C$; a small amount of red product was not characterised.

*3.4 Synthetic overview*
Generally, stable complexes of the formula $[CuLX_n(H_2O)_x]X_y(H_2O)_z$, where L = $L_A$, $L_B$ and $L_C$; X = Cl, Br, $NO_3$; n, x, y and z may have values of 0, 1 or 2, have been isolated; some of these were found to be interconvertible by the rearrangement of the ligand donor set. The conductance of all complexes determined in aqueous solution indicated the presence of 1:2 electrolytes. This behaviour may be accounted for by the formation of diaquo, octahedral $[CuL(H_2O)_2]^{2+}$ cations or square planar $[CuL]^{2+}$ cations, i.e. with no axial ligands and the non-coordinating anions providing the charge balance. Structure assignment, in terms of coordination of the anions, was achieved primarily on the balance of IR spectroscopy. Magnetic moments (Table 2) indicate normal behaviour for these $d^9$ systems. This study demonstrates that it is possible to form tetragonally distorted octahedral copper(II) complexes with the sterically congested $L_A$, $L_B$ and $L_C$ isomeric macrocycles with eight peripheral methyl groups, in particular with smaller anions. Thus, complexes with $L_A$, having four equatorial methyl groups, allowed axial coordination of all anions investigated in this study. The diaxial-diequatorial arrangement of the methyl substituents in $L_B$ precluded coordination of nitrate. By contract, $L_C$, having three equatorial methyl groups allowed the coordination of one nitrate anion only.

*3.5 Fungitoxicity study*

The antifungal activities of the isomeric macrocycles and some of their complexes are summarised in Table 4.

**TABLE 4.** *In vitro* antifungal activities of the macrocyclic ligands and their complexes

| | % inhibition of mycelial growth | | |
|---|---|---|---|
| | *Alternaria alternata* | *Carvularia lunata* | *Macrophomina phaseolina* |
| $L_A$ | 27.8 | 11.4 | 14.6 |
| $[CuL_A(NO_3)_2].H_2O$ | 15.6 | 3.9 | 11.5 |
| $[CuL_ACl_2].H_2O$ | 15.8 | 2.8 | 13.3 |
| $[CuL_ABr_2].H_2O$ | 17.6 | 5.0 | 10.0 |
| $L_B$ | 25.9 | 9.9 | 13.5 |
| $[CuL_B(H_2O)_2](NO_3)_2$ | 3.7 | 2.8 | 11.1 |
| $[CuL_B]Cl_2.2H_2O$ | 6.5 | 2.2 | 10.0 |
| $[CuL_BCl_2].H_2O$ | 6.5 | 2.8 | 10.0 |
| $[CuL_B]Br_2.2H_2O$ | 6.6 | 2.8 | 4.4 |
| $[CuL_BBr_2].H_2O$ | 6.6 | 2.1 | 3.3 |
| $L_C$ | 25.0 | 12.8 | 16.7 |
| $[CuLC(H_2O)(NO_3)]NO_3$ | 6.5 | 9.9 | 15.6 |
| $[CuL_C]Cl_2$ | 12.0 | 14.6 | 13.5 |
| $[CuL_CCl_2].2H_2O$ | 11.1 | 14.2 | 13.3 |
| $[CuL_CBr_2].2H_2O$ | 10.2 | 5.0 | 12.2 |

Screens have been conducted against three selective phytopathogenic fungi: i) *Alternaria alternata*, ii) *Curvalaria lunata*, and iii) *Macrophomina phaseolina*. The activities of the three ligands and their complexes against *Alternaria alternata* are greater than those against the other two fungi. The activities of the three macrocycles were similar and were found to decrease upon coordination to copper(II).

The fungitoxicities are generally lower that those of related sulfur-containing Schiff bases and their complexes [18], however, it is noteworthy that the decrease in activity upon coordination of the respective ligands is less in the present study.

### Acknowledgments

Financial support from the Third World Academy of Sciences (TWAS), Trieste, Italy under grant No. 93-183 RG-183 RG/CHE/AS is gratefully acknowledged. The Australian Research Council is thanked for support of the X-ray facility.

### References

1 N.F. Curtis, D.A. Swann, T.N. Waters and I.E. Maxwell, *J. Am. Chem. Soc.*, **91** (1969) 4588.
2 T. Ito and D.H. Busch, *Inorg. Chem.*, **13** (1974) 1770.
3 D.A. Swann, T.N. Waters and N.F. Curtis, *J. Chem. Soc., Dalton Trans* (1972) 1115.
4 R. Bembi, S.M. Sondhi, A.K. Singh, A.K. Jhanjii, T.G. Roy, J.W. Lown and R.G. Ball, *Bull. Chem. Soc. Jpn*, **62** (1989) 3701.
5 T.G. Roy, Ph.D. Thesis, University of Roorkee, Roorkee, U.P., India, 1987.
6 R. Bembi, M.G.B. Drew, R. Singh and T.G. Roy, *Inorg. Chem.*, **30** (1991) 1403.
7 *teXsan, Single Crystal structure analysis software*, Version 1.6 (1993), Molecular Structure Corporation, The Woodlands, TX, USA.
8 N. Walker and D. Stuart, *Acta Crystallogr.*, *A***39** (1983) 158.

9 C.K. Johnson, ORTEP. Report ORNL-5138 (1976), Oak Ridge National Laboratory, TN, USA.
10 R.W. Hay, D.A. House and R. Bembi, *J. Chem. Soc., Dalton Trans* (1984) 1927.
11 R.T. Conley, Infrared Spectroscopy (1966), Allen and Beacon Inc., Boston MA, USA, Ch 5.
12 K. Nakamoto, Infrared spectra of inorganic and coordination compounds (1963), John Wiley, New York, NY, USA.
13 C. Bianchini, L. Fabbrizzi, P. Paoletti and A.B.P. Lever, *Inorg. Chem.* **14** (1975) 197.
14 R. Bembi, R. Singh, S. Aftab, T.G. Roy and A.K. Jhanjii, *J. Coord. Chem.* **14** (1985) 119.
15 T.-J. Lee, T.-Y. Lee, W.-B. Juang and C.-S. Chung, *Acta Crystallogr.* *C***41** (1985) 1596.
16 B. Bosnich, C.K. Poon and M.L. Tobe, *Inorg. Chem.* **4** (1965) 1102.
17 T.W. Hambley, *J. Chem. Soc., Dalton Trans* (1986) 565.
18 S.K.S. Hazari, B.K. Dey and B. Ganguli, unpublished results.

**Received: June 16, 1997 - Accepted: June 30, 1997 - Received in revised camera-ready format: July 1, 1997**

